# Optimization and Comparative Evaluation of Green Extraction Techniques for Polyphenol Recovery from *Aronia melanocarpa* By-Products

**DOI:** 10.3390/foods15162853

**Published:** 2026-08-15

**Authors:** Georgios Triantafyllou, Vassilis Athanasiadis, Dimitrios Kalompatsios, Stavros I. Lalas, Paraskevi Mitlianga

**Affiliations:** 1 Department of Chemical Engineering, University of Western Macedonia, 50100 Kozani, Greece; dchemeng00041@uowm.gr; 2 Department of Food Science and Nutrition, University of Thessaly, Terma N. Temponera Street, 43100 Karditsa, Greece; dkalompatsios@uth.gr (D.K.); slalas@uth.gr (S.I.L.)

**Keywords:** aronia pomace, green chemistry, pulsed electric field extraction, pressurized liquid extraction, ultrasound-assisted extraction, response surface methodology

## Abstract

Due to its high content of bioactive constituents and associated health benefits, *Aronia melanocarpa* is considered a superfood, and its consumption has increased substantially in recent years. This growing demand has led to the generation of large quantities of processed by-products, which remain rich in valuable phytochemicals and require sustainable utilization. In this study, four extraction techniques—conventional solvent extraction (CSE), pressurized liquid extraction (PLE), pulsed electric field extraction (PEF), and ultrasound-assisted extraction (UAE)—were comparatively evaluated and optimized for the recovery of bioactive compounds from aronia pomace. A second-order polynomial model (Fit Least Squares) was applied to determine the optimal conditions for each technique. The optimized extracts exhibited distinct phytochemical profiles: total polyphenol content reached 71.5 (UAE), 70.0 (CSE), 67.0 (PLE), and 48.0 (PEF) mg GAE/g dw, while total anthocyanins were 15.5 (UAE), 13.0 (CSE), 11.0 (PEF), and 3.5 (PLE) mg CyE/g dw. Antioxidant capacity ranged from 538.0 to 800.0 µmol AAE/g dw (FRAP) and 17.0 to 39.0 mmol AAE/g dw (DPPH). HPLC analysis confirmed cyanidin-3-*O*-glucoside as the predominant compound, with concentrations of 5.0 (UAE), 4.6 (CSE), 3.2 (PEF), and 0.8 mg/g dw (PLE). Overall, ultrasound-assisted extraction under optimal conditions provided the highest recovery of bioactive constituents, highlighting its suitability for the valorization of aronia by-products.

## 1. Introduction

*Aronia melanocarpa* (Michx.) Elliott, known as black chokeberry, is a plant native to North America (USA and Canada), which is nowdays cultivated worldwide. It belongs to the *Rosaceae* family [1,2,3,4]. The aronia bush grows to a height of up to 3 m and its fruits are spherical in shape [1,5]. Due to their astringent and bitter taste, aronia fruits are not widely consumed as fresh fruits [2,3,6]. Instead, they are used in various processed forms, mainly juices but also jams, wines, natural food colorings, concentrates, jellies, nectars, and nutritional supplements [2,3,4,7,8,9]. The resulting by-products (mainly pomace), although rich in bioactive compounds, remain largely unexploited due to limited innovative utilization processes and are often either landfilled or converted into products of low economic value, such as animal feed [9,10]. Due to their high content of valuable compounds, aronia by-products have recently attracted increasing attention as raw materials for the recovery of bioactive compounds. Their utilization through appropriate extraction processes can transform these by-products into value-added products, improving the sustainability and economic efficiency of aronia processing industries. For example, rich polyphenol extracts recovered from aronia pomace can be used as natural antioxidants and colorants in food or as functional ingredients in nutritional formulations.

Aronia fruits and by-products are rich in bioactive compounds of various polyphenols such as anthocyanins, proanthocyanidins, tannins, flavonols, flavanols, and phenolic acids [2,3,6,9,11]. Polyphenols are important bioactive compounds due to their antioxidant properties, which allow the binding of free radicals, chelate metal ions, and inhibit oxidative chain reactions [12]. Aronia has been observed to exhibit the highest total polyphenol content compared to other berries [2], which typically ranges from 20 to 80 mg/g dry weight [5,13], although higher values up to 197 mg/g have occasionally been observed [7]. Anthocyanins are water-soluble anthocyanidin glycosides, present in the skin of the fruit and responsible for its purple, blue, red and black color [14], with the main anthocyanins being cyanidin-3-*O*-galactoside and cyanidin-3-*O*-arabinoside [13,15]. The main phenolic acids are chlorogenic and neochlorogenic acid [13]. Moreover, the proanthocyanidins found in the aronia plant are abundant compared to many other plants [16].

Aronia fruits and their by-products also contain carbohydrates, organic acids, proteins, fiber, essential minerals, fatty acids, carotenoids, and essential oils [3,5,6,7]. Both extracts and compounds extracted from aronia are used widely in the food, pharmaceutical and cosmetic sectors due to their positive health effects, including anticancer, antioxidant, antimicrobial, anti-inflammatory, antiviral, antidiabetic and anti-aging properties [1,2,7]. They also exhibit gastroprotective, cardioprotective, hepatoprotective, and immunomodulatory properties [2,3,4]. Moreover, aronia has been found to demonstrate positive effects on hypertension, hyperlipidemia and hypercholesterolemia [13].

Lately, the global interest in healthy foods has increased, leading to a global aronia fruits market valued at nearly $780 million in 2021 and projected to reach approximately $1.3 billion in 2028 [10,13]. Consequently, larger quantities of aronia by-products are expected to be produced, thus creating the need for their sustainable management. Therefore, researchers have focused on developing efficient extraction methodologies for the recovery of bioactive compounds using green techniques, which aim to replace conventional extraction methods associated with negative environmental impacts [17].

Ultrasound-assisted extraction (UAE), pulsed electric field extraction (PEF), microwave-assisted extraction (MAE), pressurized liquid extraction (PLE), enzyme-assisted extraction (EAE) and supercritical fluid extraction (SFE) are the most widely used green extraction techniques [18]. Among these, UAE, PEF, and PLE have gained increasing attention due to their efficiency, reduced solvent consumption, and potential to enhance the recovery of bioactive compounds compared with conventional extraction methods. In the present study, conventional solvent extraction (CSE) was also evaluated as a reference method to compare the performance of emerging extraction technologies with a commonly used solvent-based extraction approach. UAE applies ultrasound energy, generating cavitation effects that promote cell disruption and enhance mass transfer, thereby facilitating the release of bioactive compounds [15]. PEF extraction applies short-duration electrical pulses that induce electroporation of cell membranes, increasing permeability and improving the release of intracellular compounds [19]. PLE utilizes heat and high pressure for the extraction of samples (solid and solvent), aiming to increase solubility, improve solvent penetration, and enhance mass transfer rate, thus improving the efficiency and speed of the extraction process [20]. Furthermore, CSE can also be used when the solvent chosen is environmentally friendly [21]. The efficiency of these extraction procedures can be optimized by adjusting critical parameters such as temperature, pressure, solvent composition and extraction time, which influence the recovery of bioactive compounds.

Several studies have investigated the extraction of bioactive compounds from aronia pomace using different extraction approaches. UAE has been applied for the extraction of anthocyanins and phenolic compounds from aronia wastes and pomace [14,22], while other studies have investigated the use of PLE, microwave-assisted extraction, and green solvents for the recovery of bioactive compounds [3,12,15]. Moreover, the optimization of extraction conditions using response surface methodology (RSM) has been applied to individual extraction techniques for aronia pomace [10,17]. Although these studies have demonstrated the potential of different extraction approaches for the recovery of bioactive compounds from aronia pomace, most studies have focused on the optimization of individual extraction techniques or specific groups of bioactive compounds. Therefore, differences in extraction conditions and experimental approaches make it difficult to directly compare the performance of different extraction technologies. From a circular economy perspective, aronia by-products can be valorized as sources of valuable compounds for the development of value-added products not only in the food sector (e.g., bakery products, confectionery products, food packaging materials) but in other related industries as well [5,6,8,23,24]. Their incorporation into different food products and other applications can contribute to the recovery and reuse of bioactive compounds that would otherwise remain unexploited [5,6,8,24]. Such upcycling strategies can reduce waste production and improve resource efficiency, contributing to more sustainable processing of aronia and other fruit products [8,24].

Therefore, this study aimed to compare CSE, UAE, PLE, and PEF extracts from aronia by-products to determine the most effective extraction method for maximizing the recovery of bioactive compounds and antioxidant capacity under the investigated conditions. Critical extraction parameters were optimized using RSM to support the potential industrial valorization of aronia pomace. In addition, partial least squares (PLS) analysis was employed to identify the most favorable extraction conditions and to enable a comparative assessment of the optimal extracts.

## 2. Materials and Methods

### 2.1. Sample Preparation

Aronia fruits were provided by a local producer in Aridaia (Almopia, Pella, Greece). After juicing, the pomace sample was collected. The samples were freeze-dried (Biobase BK-FD10P, Jinan, China) overnight. The dried samples were ground and homogenized using an electric mill and sieved through the Analysette 3 PRO device (Fritsch GmbH, Oberstein, Germany). The diameter of the selected particles was ≤200 μm, with an average diameter of 87 μm. Finally, the powder was frozen at −40 °C until further analysis.

### 2.2. Chemicals and Reagents

Gallic acid (97%), ethanol (99.8%) and Folin–Ciocalteu reagent were obtained from Panreac Co. (Barcelona, Spain). Iron(III) chloride hexahydrate (97%) and methanol were purchased from Merck (Darmstadt, Germany). Hydrochloric acid (37%), 2,4,6-tris(2-pyridyl)-*s*-triazine (TPTZ) (≥98%), L-ascorbic acid, 2,2-diphenyl-1-picrylhydrazyl (DPPH) and all chemical standards for the HPLC determination (with ≥97% purity) were obtained from Sigma-Aldrich (Darmstadt, Germany). Anhydrous sodium carbonate was purchased from Penta (Prague, Czech Republic).

### 2.3. Extraction Techniques

Aqueous ethanol solutions were used as extraction solvents for all extraction procedures. Ethanol concentration (0–70%, *v*/*v*) and solvent-to-solid ratio (10–50 mL/g) were selected as experimental variables according to the RSM design (Table 1). After extraction, the obtained extracts were collected and further analyzed for the determination of bioactive compounds and antioxidant activity.

#### 2.3.1. Conventional Solvent Extraction (CSE)

A Heidolph stirring hotplate (Heidolph Instruments GmbH & Co. KG, Schwabach, Germany) was used to conduct the stirring process for the extraction. The conditions that remained constant in this technique were the stirring speed (500 rpm) and the magnetic stir bar (25 mm). The ethanol concentration, solvent-to-solid ratio, extraction time, and temperature were varied according to the experimental design (Table 1), while the stirring conditions remained constant throughout all experiments.

#### 2.3.2. Ultrasound-Assisted Extraction (UAE)

The ultrasound-assisted extraction procedure was conducted with an Elmasonic P70H ultrasonic bath (Elma Schmidbauer GmbH, Singen, Germany). Aqueous ethanol solutions were used as extraction solvents, while ethanol concentration, solvent-to-solid ratio, and extraction time were defined according to the experimental design (Table 1). Ultrasonic frequency (37 kHz) and extraction temperature (40 °C) were kept constant among all experiments, while the ultrasonic mode was set to pulse mode. The term “pulse mode” refers to a predefined operating mode of the ultrasonic bath and does not correspond to a specific numerical pulse duration.

#### 2.3.3. Pressurized Liquid Extraction (PLE)

A pressurized liquid extraction system (Fluid Management Systems, Inc., Watertown, MA, USA) was used for the extraction procedure using aqueous ethanol solutions as extraction solvents. The extraction pressure was kept constant at 11.72 MPa, while the ethanol concentration, solvent-to-solid ratio, extraction time, and temperature were varied according to the experimental design (Table 1).

#### 2.3.4. Pulsed Electric Field (PEF) Extraction

A digital oscilloscope (Rigol DS1052E, Beaverton, OR, USA), a mode/arbitrary waveform generator (UPG100, ELV Elektronik AG, Leer, Germany), a high-voltage power generator, and two custom stainless-steel chambers (Val-Electronic, Athens, Greece) were used. Aqueous ethanol solutions were used as extraction solvents, while ethanol concentration, solvent-to-solid ratio, and extraction time were defined according to the experimental design. The sample was treated by applying pulsed electric fields between the stainless-steel chambers under the experimental conditions defined by the RSM design. The pulse period (1000 μs) and pulse duration (100 μs) were kept constant among all experiments.

### 2.4. Experimental Design and Optimization (RSM)

A custom response surface methodology (RSM) design was constructed to evaluate the combined effects of extraction technique and processing parameters on the recovery of bioactive compounds from aronia. The design included one 4-level categorical factor corresponding to the extraction technique (X1: CSE, PEF, PLE, UAE) and four continuous factors: ethanol concentration (X2: 0–70%), solvent-to-solid ratio (X3: 10–50 mL/g), and extraction time (X4: 5–35 min). The fifth factor (X5) represented a technique-specific process-intensity parameter, treated as a continuous variable and coded at three levels (−1, 0, +1). Because each extraction technique operates under a different physical principle, X5 was defined separately for each method while maintaining a unified coded scale. Specifically, for CSE and PLE, X5 corresponded to extraction temperature, with real levels of 20, 50, and 80 °C for CSE and 40, 100, and 160 °C for PLE. For PEF, X5 corresponded to the electric field strength, with real values of 0.6, 0.8, and 1.0 kV/cm. For the UAE, X5 corresponded to ultrasonic power, expressed both as percentage amplitude (60%, 80%, 100%) and as the equivalent acoustic power output (132 W, 176 W, and 220 W, respectively). This formulation allowed all techniques to be incorporated into a single RSM model while preserving their correct physical operating ranges. However, the coded X5 values should be interpreted with caution, as they represent technique-specific process intensities rather than directly equivalent physical quantities. Therefore, comparisons based on X5 reflect the statistical modeling framework and optimization approach rather than a direct comparison of temperature, electric field strength, and ultrasonic power on an absolute physical basis.

The experimental region and factor levels were selected based on preliminary trials and constraints from the literature. Preliminary screening experiments were conducted to identify solvent compositions that ensured complete wetting of the pomace, extraction times beyond which no further increase in TPC or TAC was observed, and safe operating limits for each extraction technique (temperature for CSE/PLE, electric field strength for PEF, and ultrasonic power for UAE). These trials confirmed that the selected ranges (0–70% ethanol, 10–50 mL/g, 5–35 min, and technique-specific intensity levels) were appropriate for capturing both low- and high-efficiency extraction conditions. In addition, the chosen ranges are consistent with previously published studies on aronia pomace and similar berry matrices [1,2,14,15].

The design comprised 36 experimental runs, generated using the Custom Design platform in JMP Pro 16 (statistical software developed by SAS Institute Inc., Hong Kong, China) to estimate a second-order polynomial model including all main effects, two-factor interactions, and quadratic terms for the continuous variables. The experimental factors, coded levels, and technique-specific parameters used for each extraction method are presented in Table 1.

Design diagnostics indicated acceptable estimation and prediction capability (D-efficiency = 56.0%, G-efficiency = 69.0%, and average prediction variance = 0.475). Power analysis confirmed adequate sensitivity for detecting main effects (power > 0.95 for X2–X5) and moderate sensitivity for interaction and quadratic terms (power 0.65–0.85).

The coded model form was as follows:

(1) $$Y_{k}\;=\;\beta_{0}+\sum_{i=1}^{5}\beta_{i}X_{i}+\sum_{i=1}^{5}\beta_{ii}X_{i}^{2}+\sum_{i=1}^{5}\sum_{j=i+1}^{5}\beta_{ij}X_{i}X_{j}$$

where the independent variables are represented by *X_i_* and *X_j_*, while the predicted response is denoted as *Y_k_*. The intercept (*β*_0_) and the regression coefficients (*β_i_*, *β_ii_, β_ij_*) correspond to the linear, quadratic, and interaction effects, respectively.

### 2.5. Determination of Bioactive Compounds

#### 2.5.1. Total Polyphenol Content (TPC)

Total polyphenol content (TPC) was assessed using the Folin–Ciocalteu photometric assay [25]. Briefly, 100 μL of properly diluted sample was mixed with 100 μL of Folin–Ciocalteu reagent in a 1.5 mL Eppendorf tube. After 2 min, 800 μL of 5% *w*/*v* aqueous sodium carbonate was added to the tube and the mixture was incubated for 20 min at 40 °C. The absorbance was measured at 740 nm using a UV–Vis spectrophotometer (UV-1900i, Shimadzu, Kyoto, Japan). The total polyphenol concentration (*C*_TP_) was determined using a gallic acid standard calibration curve (10–100 mg/L with R^2^ = 0.9996). Equation (2) was used to determine the TPC as mg GAE (gallic acid equivalents) per g of dw (dry weight):

(2) $$\mathrm{TPC}\;(\mathrm{mg}\;\mathrm{GAE}/g\;\mathrm{dw})\;=\;\frac{C_{\mathrm{TP}}\;\times \;V}{w}$$

where *V* (in L) denotes the volume of the extraction medium and *w* (in g) denotes the dry weight of the sample.

#### 2.5.2. Total Anthocyanin Content (TAC)

Total anthocyanin content (TAC) was assessed using a modified spectrophotometric method in acidified ethanol [26]. Briefly, 67 μL of sample was combined with 933 μL of a 0.25 M ethanolic solution of hydrochloric acid in a 1.5 mL Eppendorf tube and the mixture was placed in a dark place for 10 min. Subsequently, the absorbance was measured at 520 nm using a UV–Vis spectrophotometer (UV-1900i, Shimadzu, Kyoto, Japan). The concentration of total pigment (*C*_TPm_) was determined from Equation (3) in CyE (cyanidin-3-*O*-glucoside equivalents):

(3) $$C_{\mathrm{TPm}}\;(\mu g\;\mathrm{CyE}/L)\;=\;\frac{A\;\times \;\mathrm{MW}\;\times \;F_{D}\;}{\varepsilon \;\times \;l}\;\times \;10^{6}$$

where *A* denotes the absorbance, MW denotes the molecular weight of cyanidin-3-*O*-glucoside (449.2 g/mol), *F*_D_ denotes the dilution factor of the sample, *ε* denotes the molar extinction coefficient of cyanidin-3-*O*-glucoside (26,900 L/(mol·cm)) and *l* denotes the path length (1 cm).

The TAC concentration (*C*_TAC_) is calculated using Equation (4):

(4) $$\mathrm{TAC}\;(\mu g\;\mathrm{CyE}/g\;\mathrm{dw})\;=\;\frac{C_{\mathrm{TPm}}\;\times \;V}{w}$$

where *C*_TPm_ is the total anthocyanin concentration in μg CyE/L, *V* (in L) denotes the volume of the extraction medium, and *w* (in g) denotes the dry weight of the sample.

#### 2.5.3. Identification of Polyphenols Through HPLC

High-performance liquid chromatography (HPLC) was used for the quantification and detection of polyphenols in the samples, as reported previously [27]. A Shimadzu SPD-M20A diode array detector (DAD) (Shimadzu Europa GmbH, Duisburg, Germany) and a Shimadzu CBM-20A liquid chromatograph (Shimadzu Europa GmbH, Duisburg, Germany) were used for the analysis of aronia pomace extracts. Separation of compounds was performed using a Phenomenex Luna C18(2) column (100 Å, 5 μm, 4.6 mm × 250 mm; Phenomenex Inc., Torrance, CA, USA) and the column temperature was maintained at 40 °C. The mobile phase consisted of 0.5% aqueous formic acid (A) and 0.5% formic acid in acetonitrile/water at a ratio of 3:2 (B). The gradient program started at 0 to 40% B, continued for 10 min at 50% B, then for another 10 min at 70% B and then remained constant for 10 min. Identification was carried out by comparing the retention time and absorption spectrum with those of the standards and quantification was carried out via calibration curves (0–50 mg/L). The flow rate of the mobile phase was 1 mL/min.

### 2.6. Determination of Antioxidant Activity

#### 2.6.1. Antiradical Activity Assay

The free radical scavenging activity of the extracts was evaluated via the DPPH radical scavenging assay [28]. Briefly, 25 μL of appropriately diluted sample was mixed with 975 μL of 100 μM of DPPH solution in methanol in a 1.5 mL Eppendorf tube. The mixture was incubated for 30 min in the dark at ambient temperature, and the absorbance of the mixture was measured at 515 nm with a UV–Vis spectrophotometer (UV-1900i, Shimadzu, Kyoto, Japan). A blank sample with DPPH solution and methanol was used at the same wavelength and the absorbance was recorded. The percentage of inhibition was determined using Equation (5):

(5) $$I\mathrm{nhibition}\;\left(\%\right)\;=\;\frac{A_{515\left(i\right)\;}-\;A_{515\left(f\right)}}{A_{515\left(i\right)}}\;\times \;100$$

For the determination of *A*_AR_ (antiradical activity) an ascorbic acid calibration curve was used. The *A*_AR_ was expressed in mmol ascorbic acid equivalents (AAE) per g of dw (dry weight), using Equation (6):

(6) $$A_{\mathrm{AR}}\;(\mathrm{mmol}\;\mathrm{AAE}/g\;dw)\;=\;\frac{C_{AA}\;\times \;V}{w}$$

where *V* (in L) denotes the volume of the extraction medium and *w* (in g) denotes the dry weight of the sample.

#### 2.6.2. Ferric-Reducing Antioxidant Power Assay

The total antioxidant capacity was evaluated via the ferric-reducing antioxidant power (FRAP) assay [29]. Briefly, 50 μL of appropriately diluted sample was mixed with 50 μL of 4 mM FeCl_3_ solution (prepared in 0.05 M HCl) in a 1.5 mL Eppendorf tube. The mixture was incubated at 37 °C for 30 min, then 900 μL of 2,4,6-tripyridyl-s-triazine (TPTZ) solution (1 mM in 0.05 M HCl) was added to the tube and the mixture was vortexed. The absorbance was measured at 620 nm with a UV–Vis spectrophotometer (UV-1900i, Shimadzu, Kyoto, Japan). The ferric-reducing power (*P*_R_) was determined using a calibration curve of ascorbic acid at a concentration range from 50 to 500 μM (*C*_AA_). The *P*_R_ was expressed in μmol ascorbic acid equivalents (AAE) per g dw, using Equation (7):

(7) $$P_{R}\;(\mu \mathrm{mol}\;\mathrm{AAE}/g\;\mathrm{dw})\;=\;\frac{C_{AA}\;\times \;V}{w}$$

where *V* (in L) denotes the volume of the extraction medium and *w* (in g) denotes the dry weight of the sample.

### 2.7. Colorimetric Analysis

Τhe CIELAB coordinates (L*, a*, and b*) of the samples were measured with a Lovibond CAM-System 500 colorimeter (The Tintometer Ltd., Amesbury, UK) to determine the color. Where L* represents the lightness of each color, a* quantifies the degree of redness (positive values) or greenness (negative values) and b* indicates the hue of the color with a tendency toward yellowness (positive values) or blueness (negative values). Chroma (C*_ab_) represents the saturation of the color, while hue angle (*h*°*_ab_*) describes the dominant color perceived.

(8) $$C_{ab}^{*}=\sqrt{{{(a}^{*})}^{2}+{{(b}^{*})}^{2}}$$

(9) $$h_{ab}^{o}=\arctan\left(\frac{b^{*}}{a^{*}}\right)$$

### 2.8. Statistical Analysis

All statistical analyses were performed using JMP Pro 16 (SAS Institute Inc.). Experimental data were fitted to second-order polynomial models using the Fit Least Squares platform. Model adequacy was evaluated through ANOVA, coefficient significance (*p* < 0.05), and diagnostic plots (residuals, normality, leverage). Model performance was assessed using the coefficient of determination (R^2^), the adjusted coefficient of determination (adjusted R^2^), the predicted residual error sum of squares coefficient of determination (PRESS R^2^) and the root mean square error (RMSE).

A partial least squares (PLS) model was additionally developed to explore multivariate relationships among predictors and responses (TPC, TAC, FRAP, DPPH) for each extraction technique. Variable importance was assessed using variable importance in projection (VIP) scores. Multi-response optimization was performed using the Prediction Profiler and desirability functions to identify the optimal extraction conditions for each technique.

## 3. Results and Discussion

### 3.1. Green Extraction Techniques

Three green extraction techniques (PLE, PEF and UAE) and one conventional method (CSE) were evaluated for the recovery of bioactive compounds. Green extraction techniques are increasingly investigated as alternatives to conventional extraction methods due to their potential to reduce environmental impact and promote the efficient recovery of valuable bioactive compounds [18]. Experimental designs were developed using the Fit Least Squares platform to optimize the extraction parameters of each technique. All extracts were analyzed for TPC, TAC, FRAP antioxidant capacity, DPPH free radical scavenging activity and their color. All optimization experiments were performed in two independent extraction replicates, and each analytical determination was conducted in triplicate. Subsequently, the optimal conditions of each extraction technique were experimentally verified once more, and the experimental results were in agreement with the values predicted by the model. The use of optimization approaches is particularly important for assisted extraction techniques, as the efficiency and selectivity of bioactive compound recovery are strongly influenced by the operating conditions applied during the extraction process [18,20,21]. The optimization of parameters such as temperature, extraction time and solvent ratio of extraction is crucial to reduce the energy of the process and to obtain enhanced extracts. It is generally accepted that the increase in temperature exhibits favorable effect on extraction processes, since temperature increases solubility and improves the diffusion coefficients. However, it has been observed that phenolic compounds can degrade when the temperature is elevated [3,14,22].

The parameters under investigation (X1–X5) are presented in Table 1. These include extraction method, solvent composition, solvent-to-solid ratio, extraction time, and process intensity (temperature for CSE and PLE, electric field strength for PEF, and ultrasonic power for UAE).

Table 2 presents the results of TPC, TAC, FRAP and DPPH for each run. The TPC recovery ranged from 11.42 to 68.34 mg GAE/g dw, while the TAC varied from 2.11 to 14.76 mg CyE/g dw. The total antioxidant capacity ranged between 183.29 and 858.06 μmol AAE/g dw, and the free radical scavenging activity from 1.74 to 37.74 mmol AAE/g dw. The highest TPC and TAC were obtained from runs 31 (68.34 mg GAE/g dw) and 4 (14.76 mg CyE/g dw), while the maximum antioxidant capacity for FRAP and DPPH was achieved in runs 17 (858.06 μmol AAE/g dw) and 5 (37.74 mmol AAE/g dw), respectively. The variation observed in TPC and TAC among the experimental runs is also in agreement with previous studies on *Aronia melanocarpa*, where extraction conditions were found to affect the recovery of phenolic compounds and anthocyanins [3,14,22].

Results of color extraction for each DP (design point) are also presented in Table 2. The L* ranges from 21.9 to 47.1, denoting significant changes in the brightness of the samples. DP 33 exhibits the maximum value, indicating the lightest sample, while the lowest appeared in DP 9 (UAE technique), corresponding to a darker product. Procedures PLE and PEF revealed higher brightness values compared to CSE and UAE in the samples. The a* exhibits a positive range from 1.7 to 38.8, indicating more red hues in the samples. The highest values were observed at DPs 19 and 8. The b* demonstrated measurements from –7 to 16.9, with the highest at DP 19. Also, the CSE procedure appears to give negative results in many DPs for the parameter b*. The C*_ab_ gives measurements from 2.6 to 42.3, with DP 19 being the highest, demonstrating that the three values (a*, b* and C*_ab_) depend on each other. Hue angle (*h°_ab_*) exhibits variation from 1.5 to 356.1, with the lowest value being present at DP 20. Most DPs revealed *h°_ab_* values close to the red areas of the color wheel. Overall, the results show that the extraction conditions and the utilized technique affect the color characteristics of the samples, altering the brightness, intensity and final shade of the color.

### 3.2. Partial Least Squares (PLS) Analysis

#### 3.2.1. Prediction Profiler

**Figure 1:**
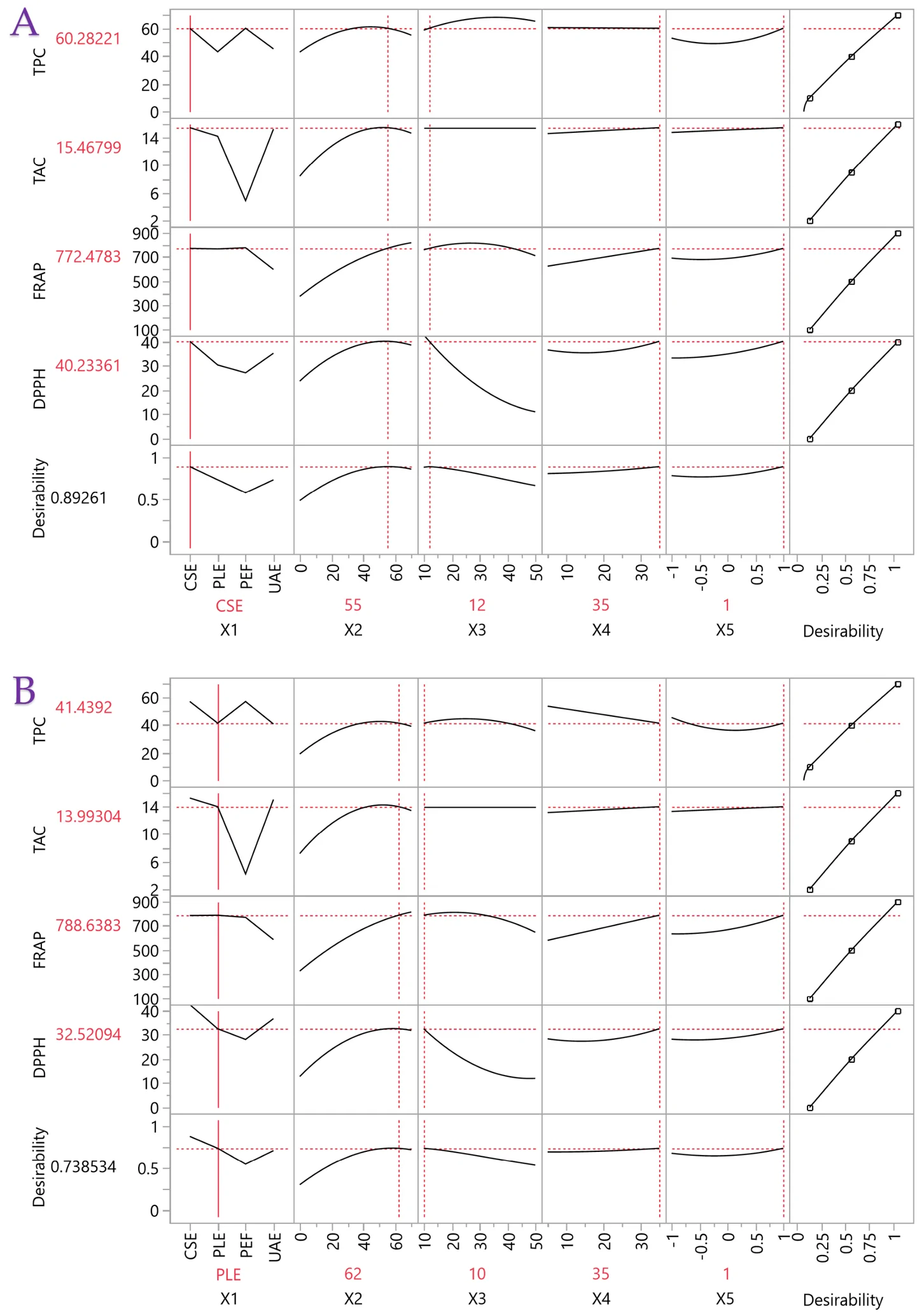

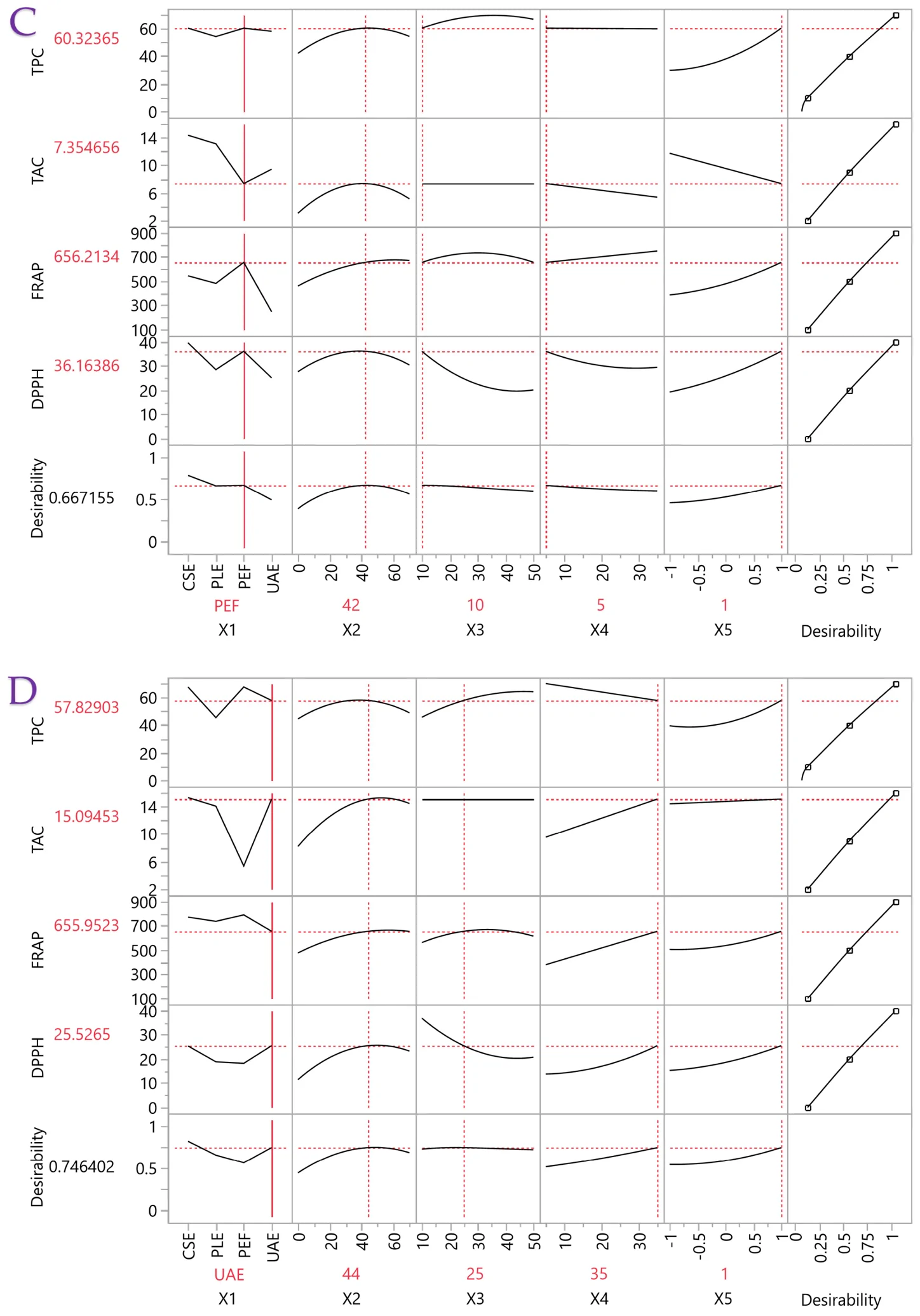
PLS-based multiple response optimization for each extraction technique. Panels (**A**–**D**) correspond to CSE, PLE, PEF, and UAE, respectively, showing predicted responses and optimal factor settings for the simultaneous maximization of antioxidant performance.

A partial least squares (PLS) analysis was applied to obtained results in order to optimize each extraction technique. The PLS-based multi-response optimization (Figure 1) performed separately for each extraction technique (CSE; PLE; PEF; UAE) revealed distinct optimal operating regions. The CSE technique produced the highest overall desirability (0.893), driven by simultaneously elevated TPC, TAC, FRAP, and DPPH values. The PLE technique yielded the highest FRAP response (~789 μmol AAE/g) but with lower TAC, resulting in moderate desirability (0.739). The PEF technique maximized TPC (~60 mg GAE/g) but exhibited substantially lower TAC and FRAP, leading to the lowest desirability (0.667). The UAE technique provided a balanced antioxidant profile with intermediate responses and desirability (0.746). These results indicate that each technique favors a different response pattern, and no single technique dominates across all antioxidant metrics. Instead, the optimal choice depends on the targeted response profile, with CSE offering the most favorable multi-response performance.

**Figure 2:**
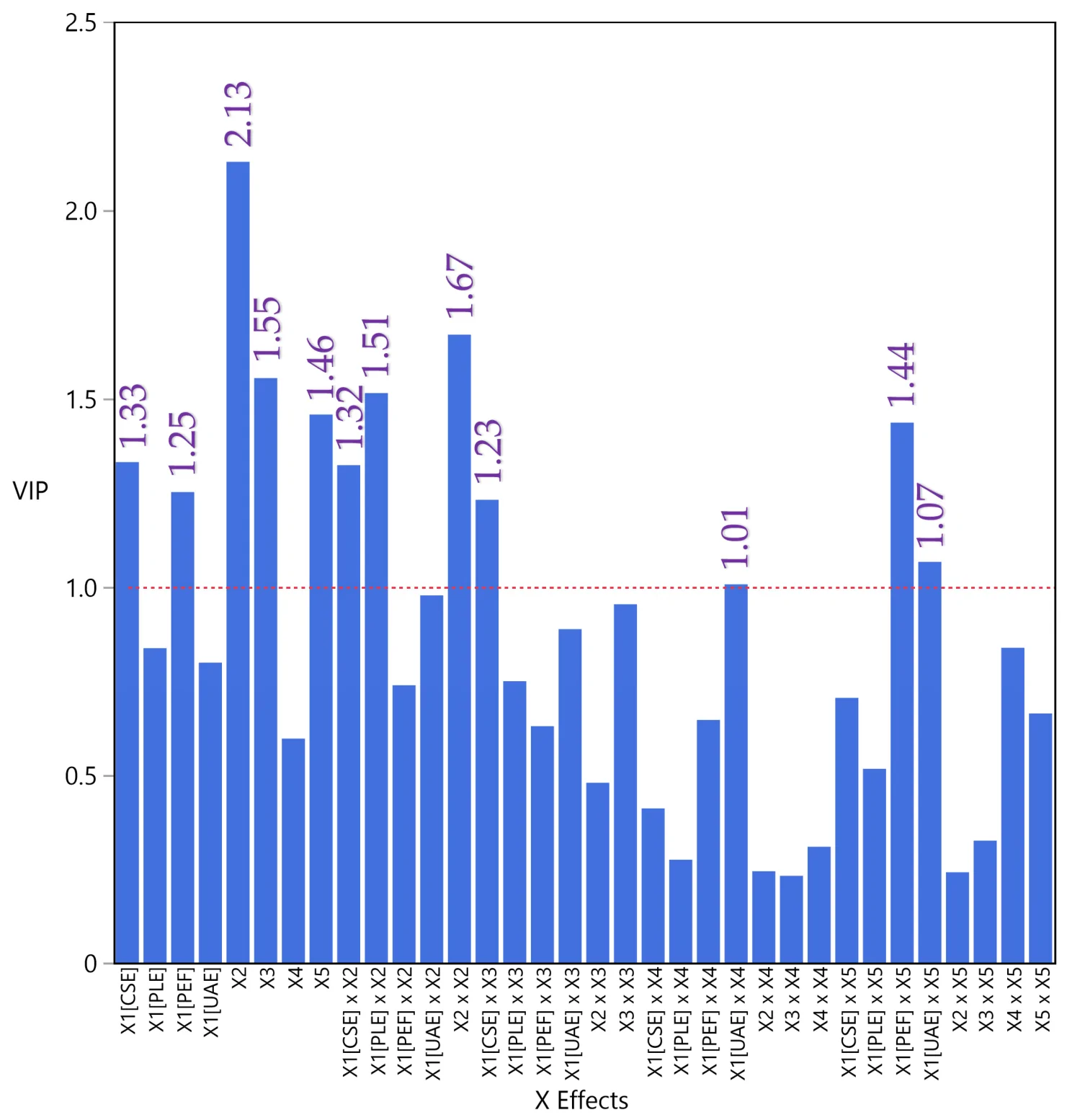
Variable importance in projection (VIP) values for the PLS model, showing the relative contribution of main effects and interaction terms. Variables with VIP > 1 were considered influential in predicting the antioxidant responses.

The VIP plot (Figure 2) indicated that X2 and X3 were the most influential predictors across the PLS models, followed by their interactions with the extraction technique (X1 × X3, X1 × X5). These findings are consistent with the interaction profiler results and explain the factor sensitivity observed in the optimization profiles.

#### 3.2.2. Model Performance and Interaction Effects

The performance of the developed PLS models and the statistical significance of the linear, quadratic, and interaction effects for all evaluated responses are summarized in Table 3. The statistical parameters obtained were used to assess the adequacy, predictive capability, and robustness of the fitted models.

The PLS models show good agreement between the experimental and the predicted values, because R^2^ and Adj R^2^ are high. From all cases, DPPH showed the strongest fit, followed by FRAP. The RMSE values were relatively low, which supports the reliability of the models in reproducing the experimental results. The PRESS R^2^ values also indicate acceptable predictive performance, although some variation is observed depending on the response. All models were highly significant (*p* < 0.0001), confirming that the relationships described are not due to random variation.

Looking at the effects of each factor, we comprehend that different responses are influenced by different combinations of variables. Some of them appear more times than others, such as X2 and X5, which means they had a more significant influence on the responses. X1 was significant mainly in TAC and FRAP, while X3 was significant mainly in DPPH and TPC.

**Figure 3:**
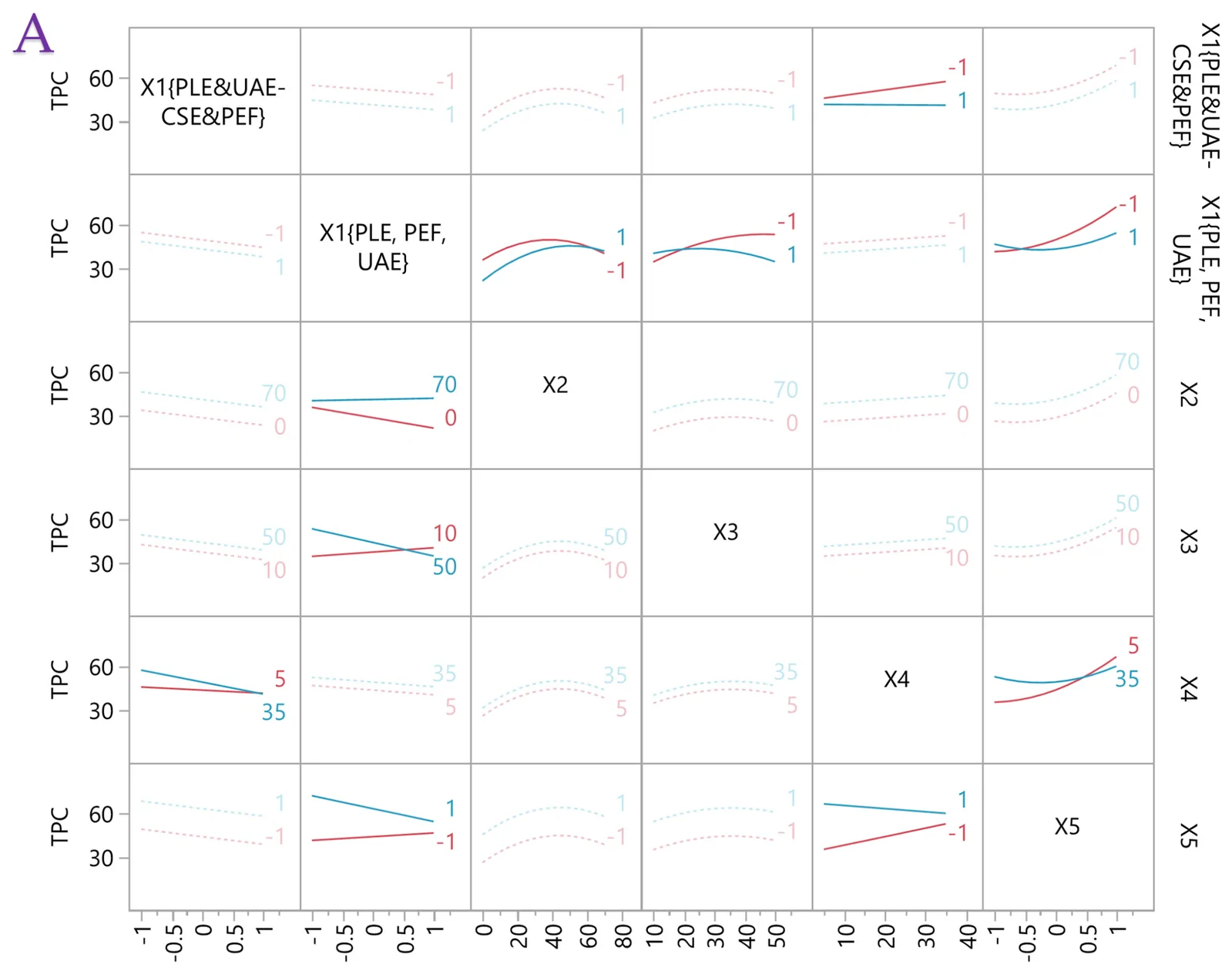

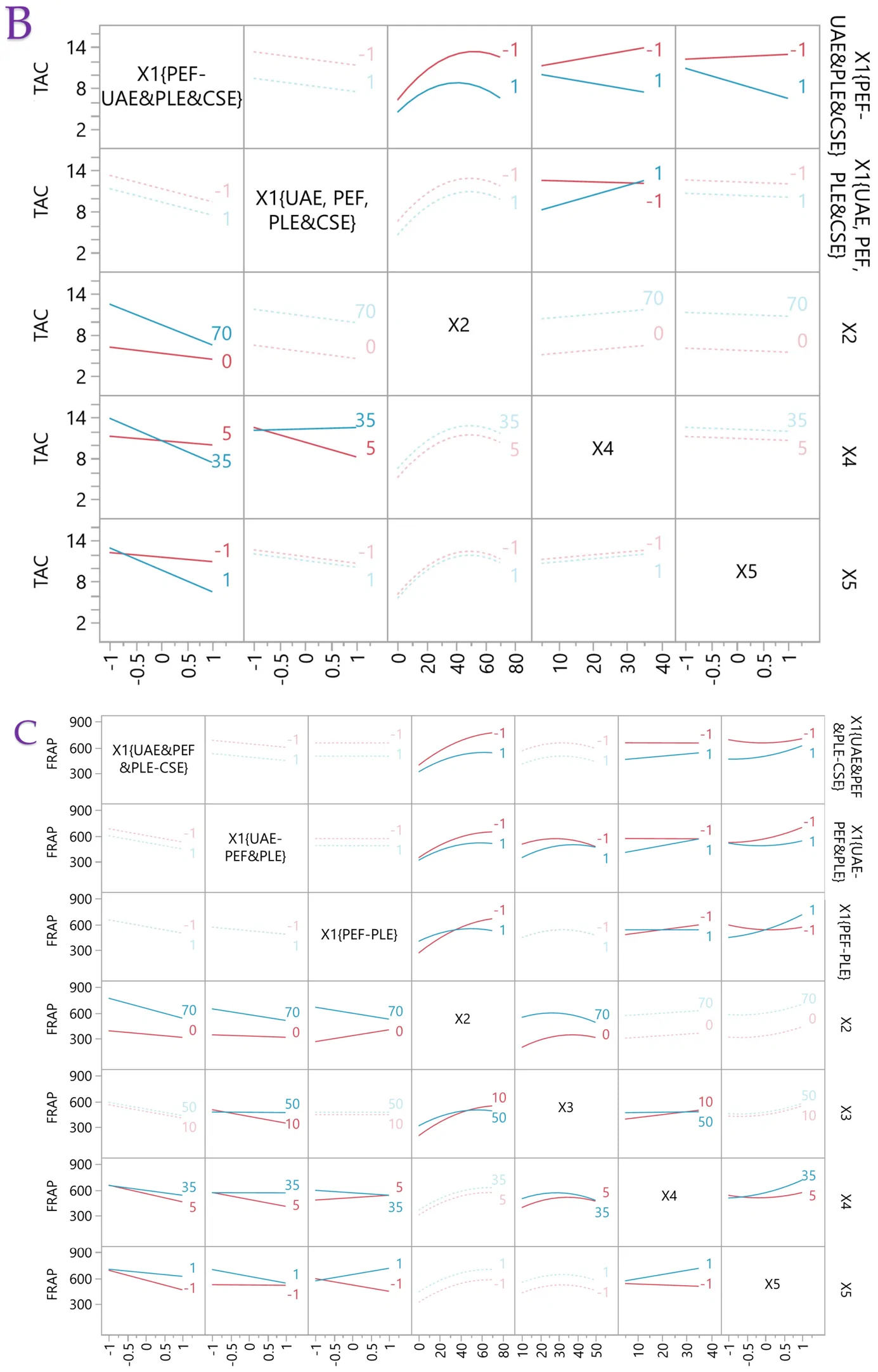

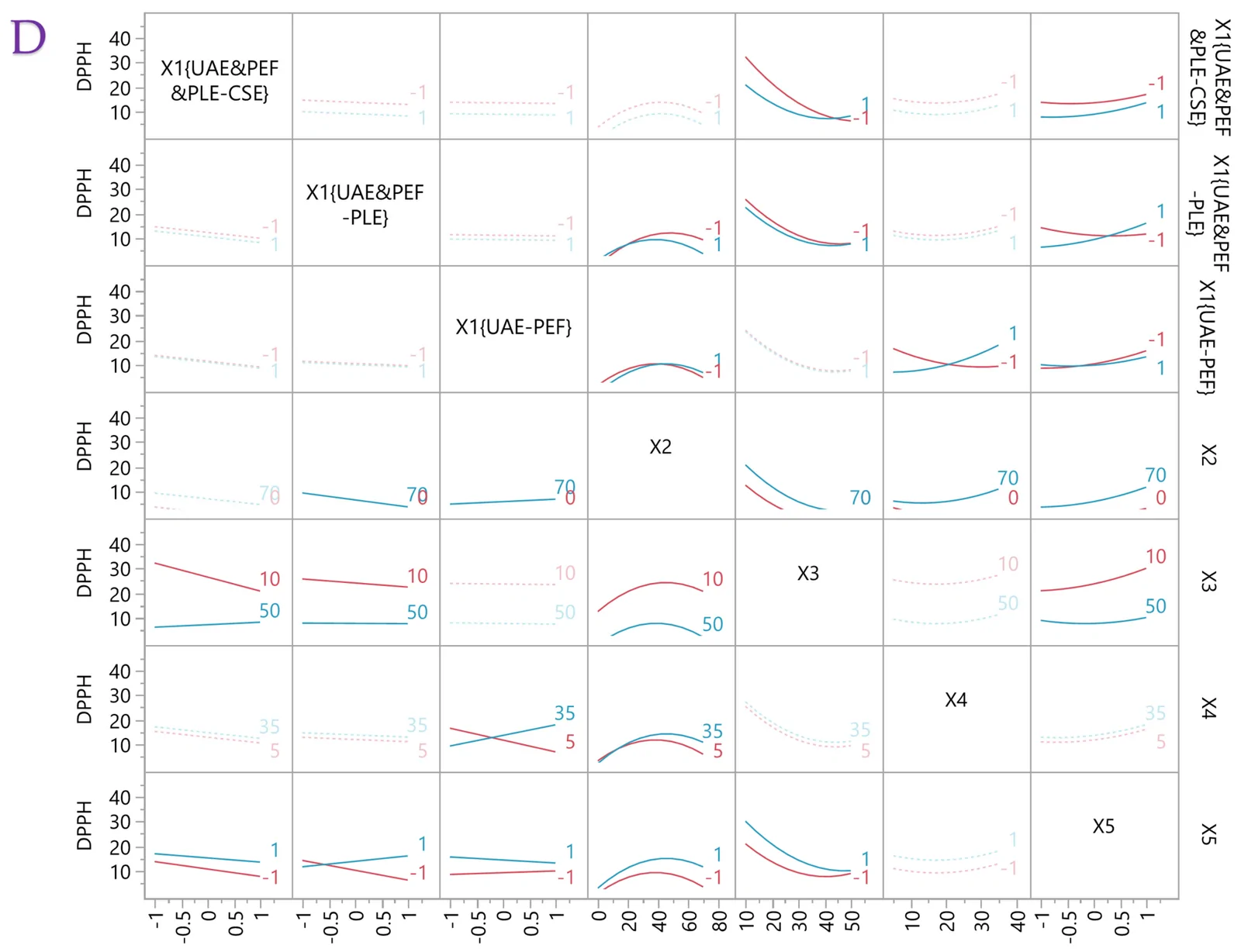
Interaction profiler matrices for all responses: (**A**) TPC, (**B**) TAC, (**C**) FRAP, and (**D**) DPPH.

The profiler visualizes pairwise factor interactions based on the fitted RSM models shown in Figure 3. Non-parallel lines indicate statistically significant interactions, whereas parallel lines indicate additive effects. TPC shows only categorical–continuous interactions (X1 × X3, X1 × X5, X4 × X5), while TAC, FRAP, and DPPH exhibit multiple interaction patterns involving both categorical and continuous factors.

### 3.3. PLS-Based Optimization and Experimental Validation

Table 4 and Table 5 present the theoretical (PLS-predicted) and experimental optima, which may differ from the average RSM predictions.

The experimentally validated optima were consistent with the PLS-predicted trends. CSE and UAE produced the highest antioxidant responses experimentally, in agreement with their high predicted desirability. UAE, despite the lower desirability, gave the highest experimental values of TPC (71.52 mg GAE/g), TAC (15.42 mg CyE/g) and FRAP (804.71 μmol AAE/g) compared to the other techniques. The largest differences between the predicted and experimental values were observed in the PLE technique. PEF showed lower TAC and moderate FRAP values, matching the PLS predictions. Colorimetric attributes varied substantially among techniques, with UAE yielding the most intense chromaticity (high a* and C*_ab_), whereas CSE and PEF produced more neutral color profiles. Overall, the agreement between predicted and measured responses confirms the robustness of the PLS models.

The TPC of the extracts ranged from 47.72 to 71.52 mg GAE/g, with the highest value observed for UAE, followed by CSE, while the lowest was obtained from PEF. Lee et al. [10] reported TPC values within a comparable range, although with limited overlap and slightly higher maximum values using response surface methodology (RSM). Likewise, Mosanu et al. [23] and Xu et al. [17] reported TPC values consistent with those obtained in the present study. In contrast, Vázquez-Espinosa et al. [22] reported significantly lower TPC values, while Kaloudi et al. [2] observed a higher value than those reported here.

The TAC of the extracts ranged from 3.56 to 15.42 mg CyE/g, with the lowest value observed for PLE and the highest for UAE. Jurendić and Ščetar [1] also reported TAC values similar to our results, while Lee et al. [10] also reported comparable values, although slightly lower than those obtained in the present study. In contrast, Ivanković et al. [24] reported significantly lower TAC values, even below the minimum value observed here. On the other hand, studies by Milutinović et al. [30] and Roda-Serrat et al. [31] reported higher TAC values than those obtained in the present work. TAC values from the literature were converted to mg CyE/g, where necessary, to facilitate comparison.

The total antioxidant capacity (TAC) of the different extraction techniques performed was approximately 538.68 to 804.71 µmol AAE/g. Tolić et al. [32] found lower concentrations, but the highest was close to our PLE value. Aprodu et al. [33], on the other hand, found a lower TAC value than ours. Kusur et al. [34] identified a range of different values, some of which were quite close to our TAC results. TAC values from the literature were converted to µmol AAE/g DM where necessary to allow for comparison.

Free radical scavenging activity was approximately 17.36 to 38.95 mmol AAE/g, with the highest values observed in PEF and CSE. Due to the use of different calibration standards in the literature (TE or %RSA) compared to the AAE units used in this study, direct numerical comparison of DPPH values is not possible. Therefore, the comparison is qualitative and based on the general range of values and the relative antioxidant behavior reported in previous studies. The experimental DPPH values obtained here fall within the broad variability observed for aronia pomace extracts, despite the unit incompatibility.

These variations, when comparing our results (TPC, TAC, FRAP, DPPH) with data from the literature, are mainly related to differences in extraction techniques and the conditions under which the extraction is carried out. Extraction parameters exhibited an important role in the recovery of bioactive compounds from aronia pomace extracts. The solvent composition, extraction time and process intensity affected the extraction efficiency by influencing the solubility and diffusion of phenolic compounds [12,18]. However, higher extraction intensity or temperature does not always lead to increased recovery since sensitive compounds may undergo degradation under severe conditions [12,15]. Among the investigated techniques, the green extraction methods showed improved performance compared with the conventional extraction due to their ability to enhance the release of bioactive compounds from the aronia pomace [3,14,18]. Therefore, the optimization of extraction conditions is essential for achieving efficient recovery of bioactive compounds from aronia pomace [10,12].

### 3.4. Principal Component Analysis (PCA) and Multivariate Component Analysis (MCA)

**Figure 4:**
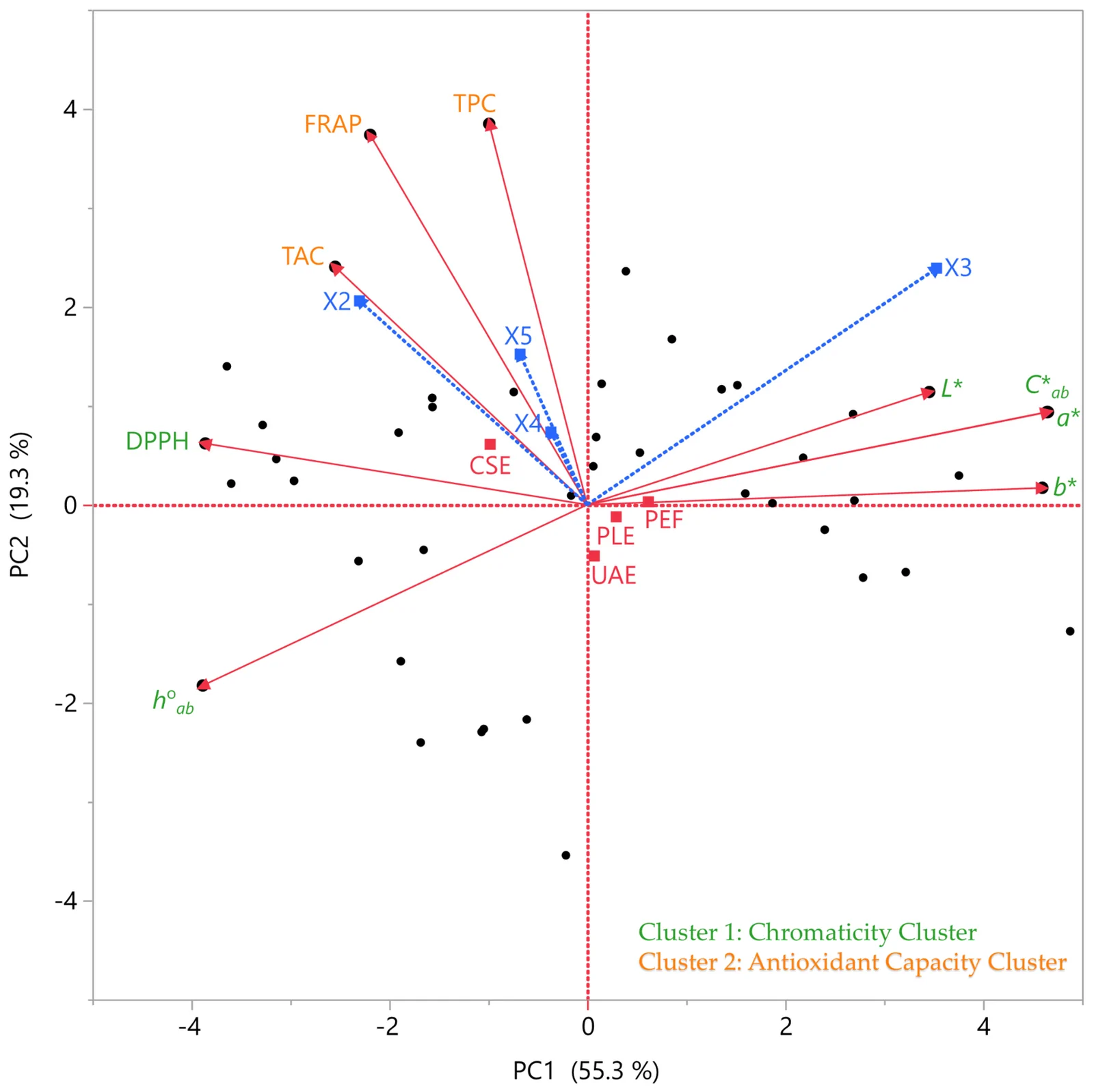
PCA biplot of antioxidant and colorimetric variables showed separation into a chromaticity cluster (PC1) and an antioxidant capacity cluster (PC2). CSE: conventional solvent extraction; PLE: pressurized liquid extraction; PEF: pulsed electric field extraction; UAE: ultrasound-assisted extraction. TPC: total phenolic content; TAC: total anthocyanin content; FRAP: ferric-reducing antioxidant power; DPPH: 2,2-diphenyl-1-picrylhydrazyl assay. L*: lightness; a*: red–green coordinate; b*: yellow–blue coordinate; C*_ab_: chroma; *h*°*_ab_*: hue angle.

Principal component analysis (PCA) revealed a clear two-dimensional structure underlying the dataset shown in Figure 4: PC1 (55.3% of the variance) was strongly associated with the colorimetric attributes L*, a*, b*, C*_ab_ and *h*°*_ab_*, together with an opposite loading of DPPH, forming a coherent chromaticity cluster. In contrast, PC2 (19.3%) was defined primarily by TPC, TAC and FRAP, representing an independent antioxidant capacity cluster. This separation indicates that color intensity and antioxidant potential constitute two distinct sources of variation across the extraction conditions. The positioning of the extraction techniques within the PCA space further highlights their differential behavior; CSE aligns more closely with the chromaticity dimension, whereas PEF shifts toward the antioxidant axis. Continuous process variables (X2–X5) contributed minimally to the PCA structure, confirming that the dominant variation arises from the analytical responses rather than the factor levels themselves.

**Figure 5:**
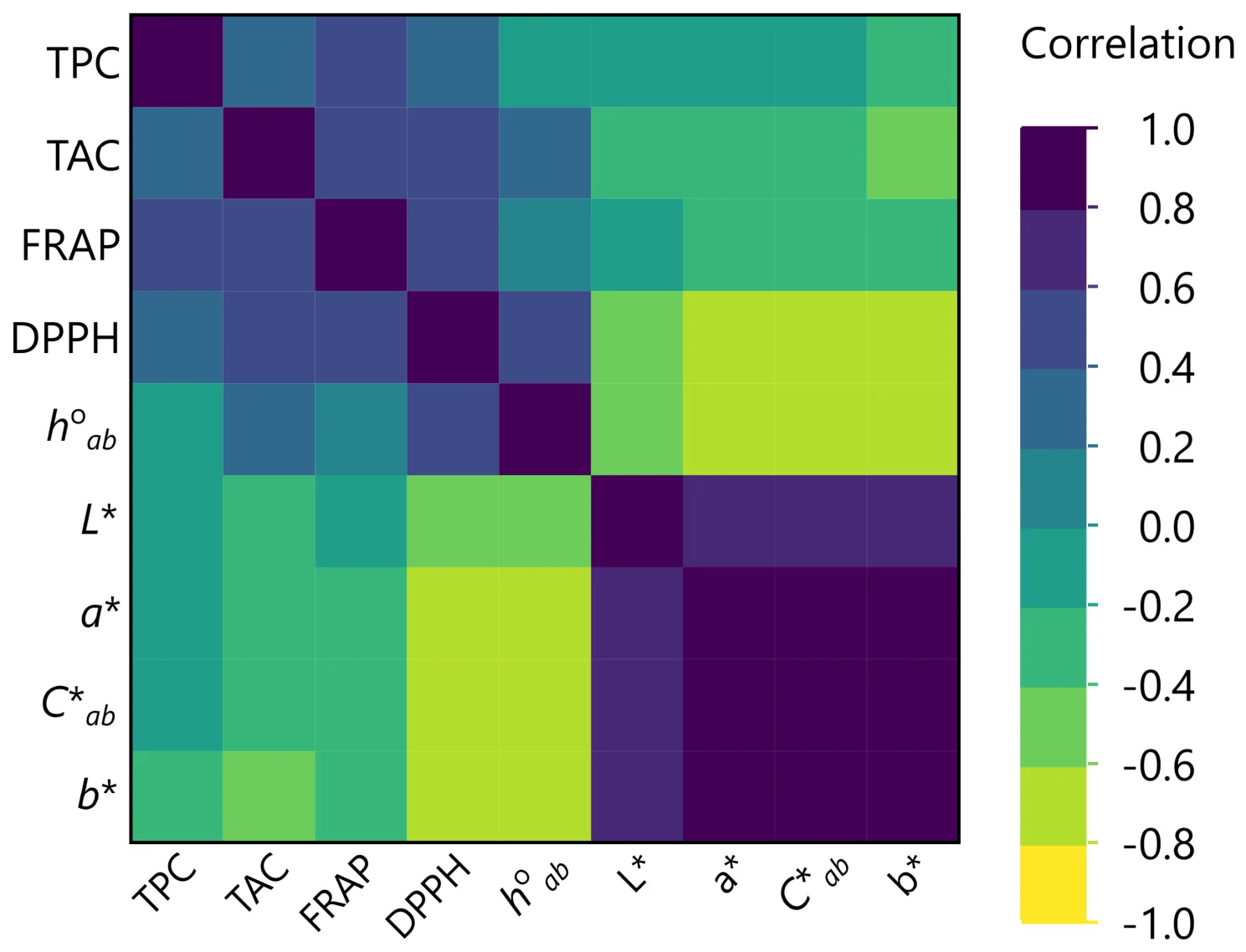
Correlation heatmap of antioxidant and colorimetric variables showing two distinct clusters: a chromaticity cluster (L*, a*, b*, C*_ab_, *h*°*_ab_*, DPPH) and an antioxidant capacity cluster (TPC, TAC, FRAP).

The correlation heatmap (Figure 5) revealed two coherent variable clusters. Colorimetric attributes (L*, a*, b*, and C*_ab_) formed a highly correlated block, with DPPH showing strong negative associations with these parameters, defining a chromaticity-related dimension. Hue angle (*h*°*_ab_*) showed a different correlation pattern, being negatively associated with the main color parameters. In contrast, TPC, TAC and FRAP clustered together, reflecting a distinct antioxidant capacity dimension. This structure is fully consistent with the PCA and variable clustering results, confirming that color intensity and antioxidant potential represent independent sources of variation across the extraction conditions.

### 3.5. Comparative Analysis of Extraction Techniques

In this subsection, a comparative analysis of the extraction techniques used in this study is presented here, evaluating their performance in terms of TPC, TAC, FRAP, and DPPH based on the results of the experimental DPs. Table 6 and Table 7 present the average performance of the techniques as derived from the RSM models over the entire experimental range. These rankings do not represent the optimal performance of each technique, but reflect the average response predicted across the experimental range studied. In contrast, the experimental and PLS-predicted optima presented in Table 4 and Table 5 represent the maximum achievable performance under specific operating conditions. Because these two approaches describe different aspects of system behavior—overall trends versus peak performance—differences between the rankings obtained from Table 4, Table 5, Table 6 and Table 7 are expected.

CSE and PEF proved to be the best procedures for the extraction of TPC, while PLE was the worst, as shown in Table 6. This observation suggests that CSE and PEF may enhance the release of phenolics through the disruption of plant cellular structures and improved mass transfer. Similar effects of extraction parameters on phenolic recovery from aronia pomace have been reported previously [12]. PEF is the best extraction technique for TAC, while CSE and PLE resulted in lower TAC concentrations compared with the other extraction techniques. The superior TAC response could be associated with PEF and is likely due to the enhancement of cell membrane permeability, facilitating the extraction of bioactive compounds while minimizing thermal degradation. Similar improvements in anthocyanin recovery using assisted extraction techniques such as UAE and PLE have been reported for aronia pomace [3]. PEF demonstrated high efficiency for both antioxidant assays; however, PLE and UAE achieved the highest values for FRAP and DPPH, respectively. CSE proved to be the least effective technique for antioxidant activity. The lower antioxidant activity observed for CSE may be related to the absence of additional mechanisms that enhance cell disruption and mass transfer, which can limit the release of antioxidant compounds from the pomace [18]. The high DPPH activity observed for UAE is most possibly related to the cavitation effect, which improves solvent penetration and facilitates the disruption of the plant cell matrix, enhancing the release of compounds with free radical scavenging activity through improved mass transfer. Similar effects of UAE on anthocyanin extraction from aronia wastes have been reported previously [14]. These observations refer to the average model behavior and do not necessarily reflect the performance of each technique under optimized conditions.

Table 7 represents the overall comparative performance of the techniques. The PEF extraction technique demonstrated the most balanced and consistently superior performance, achieving high rankings in all evaluated responses. CSE showed a strong performance only for TPC and low performance for TAC, FRAP and DPPH, suggesting that high phenolic recovery may not necessarily be related to high antioxidant activity [35]. PLE yielded the lowest TPC among all techniques, but demonstrated excellent FRAP activity compared to PEF, supporting that PLE may be suitable for applications targeting specific antioxidant compounds rather than maximum total phenolic concentration. Decreased TAC values observed for PLE may be related to the sensitivity of anthocyanins to processing conditions, including temperature and pH. Although increased temperature and pressure improve solvent diffusion and extraction efficiency, thermal exposure and unfavorable pH conditions may promote anthocyanin degradation, resulting in reduced recovery of these compounds [3,14]. However, the presence of other phenolic compounds with antioxidant properties may contribute to the elevated FRAP values observed in PLE extracts [7,10]. UAE presented a relatively balanced profile, characterized by excellent DPPH radical scavenging activity and strong TAC yield, while maintaining moderate TPC and antioxidant capacity (FRAP) values.

Our evaluation shows that the extraction efficiency is highly parameter-dependent and each technique exhibits distinct selectivity towards different classes of bioactive compounds. PEF emerged as the optimal technique due to its consistently high performance across all responses, supporting its use as an efficient and sustainable green extraction technique.

Although these rankings are based on average model predictions, the experimentally validated optima (Table 5) revealed a different performance pattern for the individual responses. In several cases, the techniques with the highest average RSM rankings were not the ones achieving the highest experimental performance at their optimal settings. This highlights the difference between global model trends and true optimal extraction behavior.

### 3.6. HPLC Analysis

The HPLC–DAD method showed excellent linearity for all quantified polyphenols (R^2^ = 0.990–0.999), with retention times ranging from 13.4 to 34.9 min and characteristic UV absorption maxima consistent with values from the literature. Sensitivity was high, with LOD values between 0.97 and 7.47 mg/L and LOQ values between 2.94 and 22.64 mg/L, confirming the suitability of the method for accurate quantification of phenolic compounds in the extracts. The DAD was set at the characteristic UV absorption maxima (λmax) of each polyphenolic compound, which are reported in Table 8.

Quantitative HPLC analysis revealed substantial differences in the polyphenolic profiles among the four extraction techniques, as presented in Table 9. UAE and CSE produced the highest concentrations of neochlorogenic acid, catechin and chlorogenic acid, whereas PLE consistently yielded the lowest levels. Cyanidin-3-*O*-glucoside, the dominant anthocyanin, was maximized under UAE (5.03 mg/g) and CSE (4.58 mg/g), with markedly lower levels in PEF and especially PLE. Rutin and quercetin derivatives were generally below the LOQ, except for quercetin-3-D-galactoside in PEF. These results demonstrate that UAE and CSE favor the extraction of both phenolic acids and anthocyanins, whereas PLE is less efficient under the tested optimal conditions.

Neochlorogenic acid (NCA) was the dominant phenolic acid detected in the extracts, a fact confirmed by previous studies that reported this compound to be the main component of phenolic acids derived from aronia pomace [1,2]. NCA concentrations measured in the present study were generally comparable to those reported in the literature, confirming its occurrence in aronia pomace. Catechin was also detected in all extraction techniques, but at higher concentrations than those reported by Ghendov-Mosanu et al. [23]. In contrast, rutin was present only in trace amounts, which was also reported by Cvetanović et al. [36]. Milutinovic et al. [30] reported a higher concentration of Quercetin-3-D-galactoside compared to our data. In contrast, the study by Jurendić and Šcetar [1] observed lower concentrations, which were nevertheless comparable to the levels achieved in our PEF extraction. Protocatechuic acid was generally either not detected or was found in negligible amounts in our research, a fact that is also confirmed by the literature review [1].

The HPLC analysis revealed a cyanidin derivative as the dominant pigment in all extracts, which is also confirmed by the literature which reports that cyanidin-3-*O*-glucoside is one of the main anthocyanin compounds in aronia pomace [1,2,15,23,30,37]. Cyanidin-3-*O*-glucoside was consistent with previous reports, although there were variations in its concentration between studies. These variations are mainly related to differences in extraction techniques and the conditions under which the extraction is carried out.

**Figure 6:**
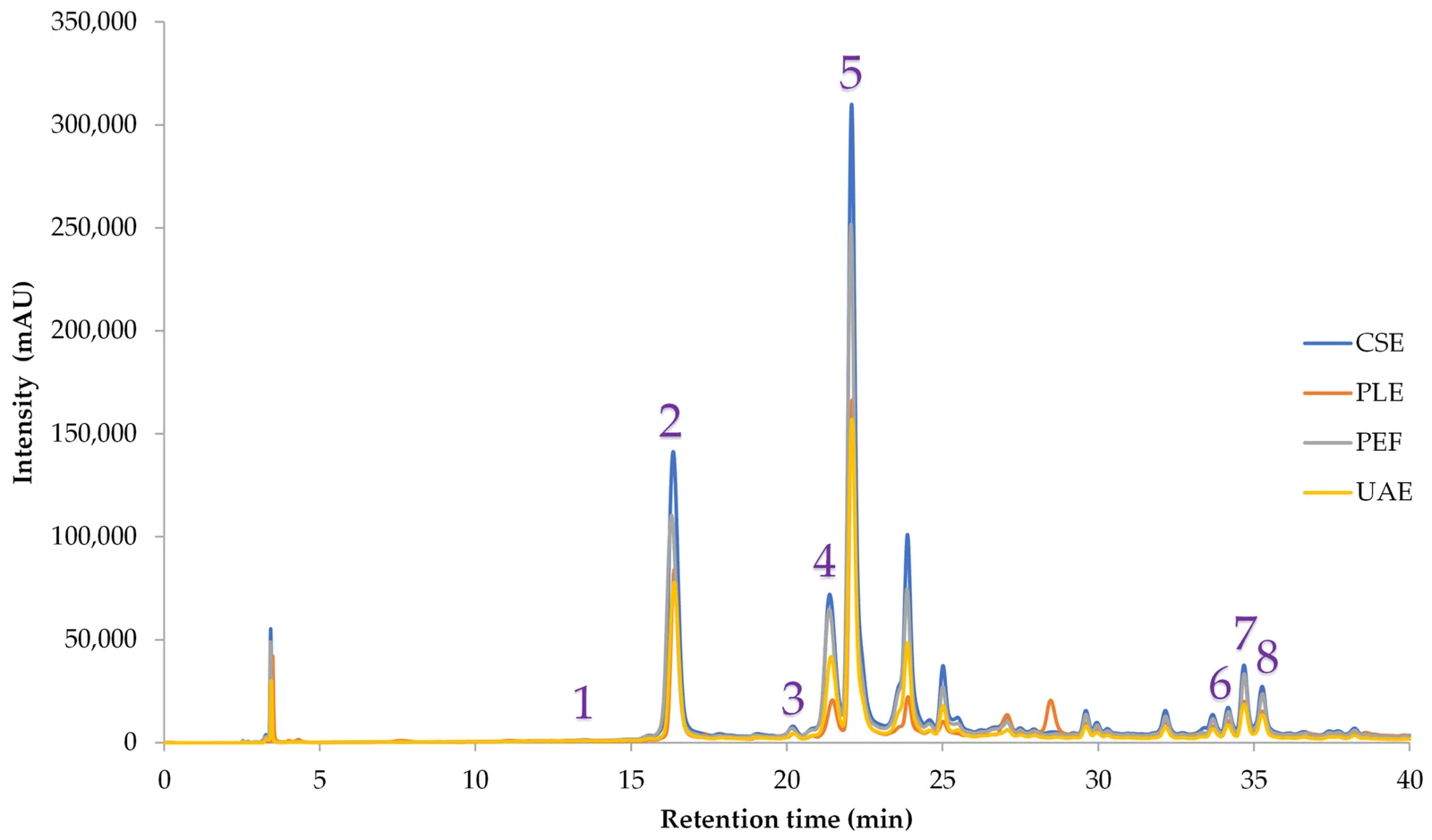
Overlay HPLC–DAD chromatograms of extracts obtained by CSE, PLE, PEF and UAE at 320 nm. Peaks correspond to the identified polyphenolic compounds: 1: Protocatechuic acid; 2: Neochlorogenic acid; 3: Catechin; 4: Cyanidin-3-*O*-glucoside; 5: Chlorogenic acid; 6: Rutin; 7: Quercetin-3-D-galactoside; 8: Quercetin-3-β-D-glucoside.

Chromatographic profiles of the four extraction techniques were overlaid at 320 nm, a wavelength representative of hydroxycinnamic acids (e.g., neochlorogenic and chlorogenic acids), which dominate the phenolic fingerprint of the extracts. Quantification of individual compounds was performed at their respective λmax. The chromatographic differences observed at 320 nm (Figure 6) are fully consistent with the quantitative HPLC results presented in Table 9.

## 4. Conclusions

Overall, our results demonstrate that both conventional and green extraction techniques can effectively recover bioactive compounds from aronia pomace. Based on the RSM models, PEF showed the most balanced performance across the entire experimental range, whereas UAE achieved the highest recovery of polyphenols, anthocyanins, and antioxidant activity under optimized conditions. These findings indicate that both PEF and UAE are suitable extraction methods of multiple bioactive constituents. Ethanol–water mixtures were confirmed as efficient and environmentally friendly solvents for all techniques.

From an industrial perspective, UAE represents a promising approach for the recovery of bioactive compounds from aronia pomace due to its efficient extraction performance and relatively simple operation. However, further studies are required to evaluate energy consumption, economic feasibility, and scalability of the optimized extraction processes. While this study evaluated these techniques on a laboratory scale, a detailed tech-no-economic assessment will be the focus of future research.

## Figures and Tables

**Table 1:** Experimental factors and coded levels.

Factor	Code	Type	Levels (Coded)	Real Levels (Example)
Method	X1	Categorical	4 levels	CSE, PEF, PLE, UAE
Ethanol concentration (%)	X2	Continuous	−1, 0, 1	0, 35, 70
Solvent-to-solid ratio (mL/g)	X3	Continuous	−1, 0, 1	10, 30, 50
Extraction time (min)	X4	Continuous	−1, 0, 1	5, 20, 35
Process intensity *	X5	Continuous	−1, 0, 1	Technique-specific parameters (*T*, *E*, *P*)

* *T* = temperature for CSE and PLE, *E* = electric field strength for PEF, and *P* = ultrasonic power UAE. The definition and corresponding actual levels of X5 for each extraction technique are provided in the text.

**Table 2:** Experimental design points and measured responses (TPC, TAC, FRAP, DPPH) and color attributes (L*, a*, b*, C*_ab_, *h°_ab_*) across all extraction techniques.

DP	Technique (X1)	X2	X3	X4	X5	TPC	TAC	FRAP	DPPH	L*	a*	b*	C*_ab_	*h°_ab_*
1	CSE	−1	0	1	1	42.73	9.92	503.38	6.89	34.6	21.0	−1.5	21.1	356.1
2	UAE	0	1	−1	−1	33.72	8.28	403.80	4.57	30.1	30.5	5.6	31.0	10.6
3	PEF	1	1	−1	0	34.57	7.66	427.64	5.05	35.3	26.3	2.5	26.4	5.5
4	CSE	0	−1	1	−1	49.57	14.76	520.12	33.98	29.1	5.4	−6.9	8.8	308.4
5	CSE	1	−1	0	1	56.97	14.19	707.03	37.74	28.6	3.5	−7.0	7.8	296.7
6	CSE	1	1	1	0	58.91	13.72	674.03	7.52	33.4	21.3	−1.7	21.4	355.4
7	UAE	−1	0	−1	1	60.87	4.54	227.05	1.74	28.6	29.1	8.3	30.3	15.8
8	CSE	−1	1	0	−1	32.62	7.26	428.95	1.98	44.4	36.5	11.9	38.4	18.1
9	UAE	0	−1	1	1	48.22	13.13	535.39	35.60	21.9	3.8	−3.5	5.2	316.7
10	PEF	−1	0	−1	−1	19.63	6.39	294.34	4.37	35.2	31.8	10.3	33.4	18.0
11	PLE	−1	0	1	−1	24.84	6.30	267.77	4.14	41.8	30.5	10.6	32.3	19.1
12	PEF	0	0	0	0	56.38	6.79	484.51	6.87	39.3	25.5	4.8	26.0	10.7
13	UAE	1	−1	−1	0	12.76	4.43	188.87	9.19	23.4	13.2	−0.9	13.3	356.0
14	UAE	1	0	1	−1	37.76	12.83	498.23	9.81	22.7	14.3	−1.5	14.4	353.9
15	PLE	0	−1	−1	−1	37.81	13.39	485.20	26.29	32.2	5.4	−3.5	6.4	326.2
16	PEF	1	0	1	1	56.18	2.11	736.08	10.07	34.5	21.8	4.6	22.3	11.5
17	CSE	1	0	−1	−1	34.41	13.83	858.06	11.27	30.2	13.5	−6.2	14.9	334.7
18	PEF	−1	−1	1	0	32.34	4.59	266.15	10.51	31.5	4.8	−2.5	5.5	332.1
19	PLE	−1	1	−1	0	11.42	5.77	226.76	1.83	45.2	38.8	16.9	42.3	23.5
20	CSE	0	1	−1	1	54.49	13.49	574.41	7.51	34.4	24.4	0.7	24.4	1.5
21	PLE	−1	−1	0	1	16.20	4.23	183.29	9.07	32.7	11.4	−1.4	11.5	352.7
22	PEF	1	−1	0	−1	34.35	10.30	374.70	7.23	23.5	3.8	−3.5	5.2	314.8
23	PLE	0	1	1	1	37.24	12.26	556.47	10.62	36.0	27.3	7.5	28.4	15.2
24	PLE	1	1	0	−1	23.34	11.86	450.51	6.47	35.0	19.2	3.0	19.4	8.8
25	CSE	0	0	0	0	51.02	13.67	651.02	11.16	32.0	16.1	−3.4	16.5	347.7
26	PEF	0	−1	−1	1	64.11	7.57	660.66	37.13	24.3	5.3	−3.3	6.4	327.0
27	CSE	−1	−1	−1	0	19.53	5.55	212.44	24.52	29.3	7.7	−5.6	9.6	323.4
28	UAE	0	0	0	0	39.08	13.62	503.66	10.83	25.9	19.2	1.4	19.3	4.0
29	PLE	1	−1	1	0	33.98	14.04	767.97	27.22	30.5	1.7	−1.9	2.6	310.4
30	UAE	−1	−1	0	−1	13.84	4.62	190.95	5.68	24.2	15.3	−1.2	15.3	355.8
31	UAE	1	1	0	1	68.34	13.80	515.19	8.15	25.9	22.1	1.2	22.1	3.0
32	PLE	1	0	−1	1	57.46	13.82	661.28	11.42	31.5	14.0	−3.0	14.5	346.3
33	PEF	0	1	1	−1	54.58	9.01	260.23	6.15	47.1	30.5	5.1	30.9	9.6
34	PLE	0	0	0	0	45.21	13.50	513.76	11.42	32.4	17.1	2.5	17.3	8.2
35	PEF	−1	1	0	1	59.44	2.85	604.24	7.51	34.1	27.1	9.8	28.8	19.9
36	UAE	−1	1	1	0	28.76	7.99	314.61	4.78	31.3	34.4	11.4	36.2	18.3

DP: design point; TPC: total polyphenol content in mg GAE/g; TAC: total anthocyanin content in mg CyE/g; FRAP: ferric-reducing antioxidant power in µmol AAE/g; DPPH: 2,2-diphenyl-1-picrylhydrazyl in mmol AAE/g. Conventional solvent extraction (CSE); pressurized liquid extraction (PLE); pulsed electric field extraction (PEF); ultrasound-assisted extraction (UAE). L*: lightness; a*: red–green coordinate, where positive values indicate redness and negative values indicate greenness; b*: yellow–blue coordinate, where positive values indicate yellowness and negative values indicate blueness; C*_ab_: chroma, representing color saturation; *h*°*_ab_*: hue angle, describing the dominant color perception.

**Table 3:** Model statistics and key effects for all responses.

Response	R^2^	Adj R^2^	RMSE	PRESS R^2^	Model F-Ratio	Model *p*-Value	Lack-of-Fit *p*-Value	Significant Main Effects	Significant Quadratic Terms	Significant Interactions
TPC	0.885	0.808	7.02	0.636	11.54	<0.0001	0.3985	X5, X2, X3	X2^2^, X3^2^, X5^2^	X4 × X5, X1 × X3, X1 × X5
TAC	0.886	0.834	1.63	0.73	16.94	<0.0001	n.r.	X2, X1	X2^2^	X1 × X2, X1 × X4, X1 × X5
FRAP	0.958	0.878	64.32	0.497	11.94	<0.0001	n.r.	X2, X1, X5	X2^2^, X3^2^	X1 × X2, X1 × X3, X1 × X5, X4 × X5
DPPH	0.984	0.954	2.22	0.775	32.92	<0.0001	n.r.	X3, X2, X5	X2^2^, X3^2^, X4^2^, X5^2^	X1 × X3, X1 × X4, X1 × X5, X2 × X3, X2 × X4, X3 × X5

n.r. = not reported.

**Table 4:** PLS-predicted optimal conditions and antioxidant responses for each extraction technique.

Technique (X1)	X2	X3	X4	X5	Predicted TPC	Predicted TAC	Predicted FRAP	Predicted DPPH	Desirability
CSE	55	12	35	1	60.28	15.47	772.48	40.23	0.893
PLE	62	10	35	1	41.44	13.99	788.64	32.52	0.739
PEF	42	10	5	1	60.32	7.35	656.21	36.16	0.667
UAE	44	25	35	1	57.83	15.09	655.95	25.53	0.746

CSE: conventional solvent extraction; PLE: pressurized liquid extraction; PEF: pulsed electric field extraction; UAE: ultrasound-assisted extraction. TPC: total phenolic content; TAC: total anthocyanin content; FRAP: ferric-reducing antioxidant power; DPPH: 2,2-diphenyl-1-picrylhydrazyl assay.

**Table 5:** Experimental responses and color attributes of extracts obtained under the optimal conditions of each extraction technique.

Technique (X1)	X2	X3	X4	X5	TPC (mg GAE/g)	TAC (mg CyE/g)	FRAP (µmol AAE/g)	DPPH (mmol AAE/g)	L*	a*	b*	C*_ab_	*h°_ab_*
CSE	55	12	35	1	70.06 ± 1.50 ^a^	13.16 ± 0.27 ^b^	772.24 ± 10.59 ^b^	34.29 ± 0.62 ^b^	31.9 ± 0.6 ^b^	1.4 ± 2.5 ^b^	–3.3 ± 2.0 ^b^	3.8 ± 2.7 ^b^	279.4 ± 29.4 ^a^
PLE	62	10	35	1	67.20 ± 1.53 ^a^	3.56 ± 0.16 ^d^	538.68 ± 14.42 ^d^	29.10 ± 1.47 ^c^	31.0 ± 0.7 ^b^	3.5 ± 1.6 ^b^	–3.0 ± 0.5 ^b^	4.7 ± 0.9 ^b^	318.1 ± 16.6 ^a^
PEF	42	10	5	1	47.72 ± 1.03 ^b^	11.23 ± 0.08 ^c^	713.03 ± 4.62 ^c^	38.95 ± 2.45 ^a^	30.2 ± 1.1 ^b^	1.7 ± 2.0 ^b^	–3.8 ± 0.5 ^b^	4.4 ± 0.5 ^b^	293.1 ± 25.8 ^a^
UAE	44	25	35	1	71.52 ± 3.11 ^a^	15.42 ± 0.38 ^a^	804.71 ± 8.37 ^a^	17.36 ± 1.21 ^d^	34.5 ± 0.7 ^a^	14.3 ± 2.0 ^a^	1.5 ± 0.9 ^a^	14.4 ± 2.0 ^a^	5.6 ± 3.3 ^b^

CSE: conventional solvent extraction; PLE: pressurized liquid extraction; PEF: pulsed electric field extraction; UAE: ultrasound-assisted extraction. TPC: total phenolic content; TAC: total anthocyanin content; FRAP: ferric-reducing antioxidant power; DPPH: 2,2-diphenyl-1-picrylhydrazyl assay. L*: lightness; a*: red–green coordinate; b*: yellow–blue coordinate; C*_ab_: chroma; *h*°*_ab_*: hue angle. Values are presented as mean ± standard deviation of three analytical replicates performed using the same extract (n = 3). Different superscript letters within the same column indicate statistically significant differences (*p* < 0.05).

**Table 6:** Technique ranking per response.

Response	1st (Best)	2nd	3rd	4th (Worst)	Comment
TPC	CSE ≈ PEF	UAE	PLE	PLE	PLE clearly weakest for TPC
TAC	PEF	UAE	PLE ≈ CSE	–	PEF clearly superior
FRAP	PEF ≈ PLE	UAE	CSE	CSE	CSE is clearly worse for FRAP
DPPH	UAE ≈ PEF	PLE	CSE	CSE	PEF & UAE dominate DPPH

CSE: conventional solvent extraction; PLE: pressurized liquid extraction; PEF: pulsed electric field extraction; UAE: ultrasound-assisted extraction. TPC: total phenolic content; TAC: total anthocyanin content; FRAP: ferric-reducing antioxidant power; DPPH: 2,2-diphenyl-1-picrylhydrazyl assay.

**Table 7:** Overall comparative performance of techniques.

Technique	TPC Performance	TAC Performance	FRAP Performance	DPPH Performance	Overall Assessment
CSE	High (≈PEF)	Moderate–low	Lowest	Lowest	Good for TPC, weak antioxidant profile overall
PEF	High (≈CSE)	Best	Best (≈PLE)	Best (≈UAE)	Most balanced and generally superior
PLE	Lowest	Moderate	Best (≈PEF)	Intermediate	Strong FRAP, weak TPC
UAE	Intermediate	Second	Intermediate	Best (≈PEF)	Very good DPPH, solid TAC, mid-range TPC/FRAP

CSE: conventional solvent extraction; PLE: pressurized liquid extraction; PEF: pulsed electric field extraction; UAE: ultrasound-assisted extraction. TPC: total phenolic content; TAC: total anthocyanin content; FRAP: ferric-reducing antioxidant power; DPPH: 2,2-diphenyl-1-picrylhydrazyl assay.

**Table 8:** Validation parameters of the HPLC–DAD method for quantification of polyphenolic compounds.

Polyphenolic Compound	Equation (Linear)	R^2^	RT (min)	UVmax (nm)	LOD (mg/L)	LOQ (mg/L)
Protocatechuic acid	*y* = 21,282.98*x* − 63,918.97	0.977	13.400	260	7.47	22.64
Neochlorogenic acid	*y* = 28,213.52*x* + 551.72	0.999	16.498	324	1.74	5.26
Catechin	*y* = 11,920.79*x* − 128.19	0.997	20.933	278	2.54	7.71
Cyanidin-3-glucoside chloride	*y* = 46,680.57*x* − 10.63	0.999	21.312	516	0.97	2.94
Chlorogenic acid	*y* = 50,320.40*x* − 23,038.36	0.994	21.947	325	3.67	11.11
Rutin	*y* = 46,365.62*x* − 31,562.74	0.997	33.777	254	2.65	8.03
Quercetin 3-D-galactoside	*y* = 41,489.69*x* − 35,577.55	0.993	34.598	257	3.96	12.00
Quercetin 3-β-D-glucoside	*y* = 45,580.75*x* + 94,644.94	0.990	34.866	256	4.83	14.65

**Table 9:** Quantified polyphenolic compounds (mg/g dw) at the optimal conditions for each extraction technique.

A/A	Polyphenolic Compound	CSE	PLE	PEF	UAE
1	Protocatechuic acid	n.d.	0.17 ± 0.01	n.d.	n.d.
2	Neochlorogenic acid	1.37 ± 0.03 ^a^	0.54 ± 0.03 ^b^	n.d.	1.48 ± 0.11 ^a^
3	Catechin	0.37 ± 0.02 ^a^	0.09 ± 0 ^c^	0.20 ± 0.01 ^b^	0.33 ± 0.02 ^a^
4	Cyanidin-3-*O*-glucoside	4.58 ± 0.21 ^b^	0.81 ± 0.05 ^d^	3.27 ± 0.07 ^c^	5.03 ± 0.28 ^a^
5	Chlorogenic acid	1.36 ± 0.05 ^b^	0.60 ± 0.04 ^d^	0.92 ± 0.03 ^c^	1.46 ± 0.03 ^a^
6	Rutin	<LOQ	<LOQ	<LOQ	<LOQ
7	Quercetin-3-D-galactoside	<LOQ	<LOQ	0.15 ± 0.01	<LOQ
8	Quercetin-3-β-D-glucoside	<LOQ	<LOQ	n.d.	<LOQ

Superscript letters (a–d) denote statistically significant differences (*p* < 0.05) in each row. n.d. = not detected; <LOQ = below limit of quantification.

## Data Availability

The original contributions presented in this study are included in the article, and further inquiries can be directed to the corresponding author.

## Abbreviations

The following abbreviations are used in this manuscript:

AAE	Ascorbic acid equivalents
CSE	Conventional Solvent Extraction
CyE	Cyanidin-3-*O*-glucoside equivalents
FRAP	Ferric-Reducing Antioxidant Power
GAE	Gallic acid equivalents
MCA	Multivariate Component Analysis
NCA	Neochlorogenic acid
PLE	Pressurized Liquid Extraction
PEF	Pulsed Electric Field extraction
PLS	Partial least squares
PCA	Principal Component Analysis
RSM	Response Surface Methodology
TPC	Total polyphenol content
TAC	Total anthocyanin content
TPTZ	2,4,6-tripyridyl-*s*-triazine
UAE	Ultrasound-assisted extraction
VIP	Variable Importance in Projection
